# Supplementary material for: Resolving the full spectrum of human genome variation using Linked-Reads
Source: Genome Res. 2019 Apr;29(4):635–45. doi: 10.1101/gr.234443.118 (PMC6442396; doi:10.1101/gr.234443.118)
Supplement: Supplemental Material [file supp_gr.234443.118_Supplemental_longranger-2.2.zip › longranger-2.2.tar.bz2!/lr2.2-public/lib/stan/cmdstan/stan/doc/api/html/classstan_1_1common_1_1recorder_1_1messages-members.html]

Stan: Member List


|  |  |
| --- | --- |
|  | Stan  2.5.0  probability, sampling & optimization |


- Main Page
- Related Pages
- Namespaces
- Classes
- Files

- Class List
- Class Index
- Class Hierarchy
- Class Members

All Classes Namespaces Files Functions Variables Typedefs Enumerations Enumerator Friends Macros Pages

- stan
- common
- recorder
- messages

stan::common::recorder::messages Member List

This is the complete list of members for stan::common::recorder::messages, including all inherited members.

|  |  |  |
| --- | --- | --- |
| is\_recording() const | stan::common::recorder::messages | inline |
| messages(std::ostream \*o, std::string prefix) | stan::common::recorder::messages | inline |
| messages(std::ostream \*o) | stan::common::recorder::messages | inline |
| operator()(const std::vector< T > &x) | stan::common::recorder::messages | inline |
| operator()(const std::string x) | stan::common::recorder::messages | inline |
| operator()() | stan::common::recorder::messages | inline |


---

[ Stan Home Page ]

*© 2011–2014,
Stan Development Team.*
